# Locking Compression Plate in Distal Femoral Intra-Articular Fractures: Our Experience

**DOI:** 10.1155/2014/372916

**Published:** 2014-07-17

**Authors:** G. N. Kiran Kumar, Gaurav Sharma, Kamran Farooque, Vijay Sharma, Ratnav Ratan, Sanjay Yadav, Devendra Lakhotia

**Affiliations:** Department of Orthopaedics, AIIMS, New Delhi 110029, India

## Abstract

*Background*. Intra-articular fractures of distal femur present a huge surgical challenge. The aim of this study is to evaluate functional outcome, fracture healing, and the complications of distal femoral intra-articular fractures using locking compression plates. *Material and Methods*. We reviewed 46 distal femoral fractures treated with distal femoral locking compression plates between 2009 to 2012. There were 36 men and 10 women with mean age of 35 years (range 20–72). More than half of the patients were of type C3 (AO classification) and had been caused by high energy trauma with associated injuries. *Results*. 2 patients were lost to follow-up. Of the remaining 44 patients, the mean follow-up period was 25 months (range 18–36). The mean time for radiological union was 12 weeks (range 10–18) except 2 patients which had gone for nonunion. At the latest follow up ROM >120° is noted in 32 patients, 90–120 in 10 patients, and 70–90 in 2 patients. 38 patients (86%) had good/excellent outcome. *Conclusion*. Use of standard lateral approach for simple intra-articular distal femoral fractures (C1) and transarticular/minimally invasive techniques for complex intra-articular fractures (C2/C3) results in improved exposure of the knee joint and better union rates with low incidence of bone grafting.

## 1. Introduction

Intra-articular fractures of distal femur present a huge surgical challenge. These fractures are difficult to treat and operative treatment is usually recommended for favorable outcome [1–4]. These are associated with high energy trauma (in the youngsters) and osteoporotic bones (in the elderly) [5] and are frequently comminuted and intra-articular.

Current generation of distal femoral locking compression plates is precontoured based on the average bony anatomy of the adult population and they form a fixed angled construct. The pull-out strength of locking screws is higher than the conventional screws and is particularly useful in osteoporotic bones. These plates are designed to apply in minimally invasive fashion to preserve local biology and avoid problems with fracture healing and infection [6, 7].

The purpose of this study was to evaluate functional outcome, fracture healing, and the complications of distal femoral intra-articular fractures using locking compression plates.

## 2. Material and Methods

We reviewed 46 consecutive distal femoral fractures treated with distal femoral locking compression plates between 2009 and 2012. AO Synthes distal femoral locking compression plate and Zimmer periarticular distal femoral locking plate were used in 19 and 27 patients, respectively. There were 36 men and 10 women with mean age of 35 years (range 20–72).

Fractures were categorized according to AO/OTA classification. Inclusion criteria were as follows: (i) type C distal femoral fractures and (ii) patients older than 18 years of age. Pathological fractures and types A and B distal femoral fractures were excluded from the study.

10 patients had intra-articular fractures involving both condyles (33C-1), 12 patients had metaphyseal comminution (33C-2), and 24 patients had articular comminution (33C-3). Definitive fracture fixation was performed in 33 patients within 7 days. 13 patients with multiple injuries and open fractures were treated within 3 wks after temporary application of external fixator. Preoperative AP and lateral radiographs of affected knee with femur were obtained. CT was obtained in 30 patients.

**Figure 1:**
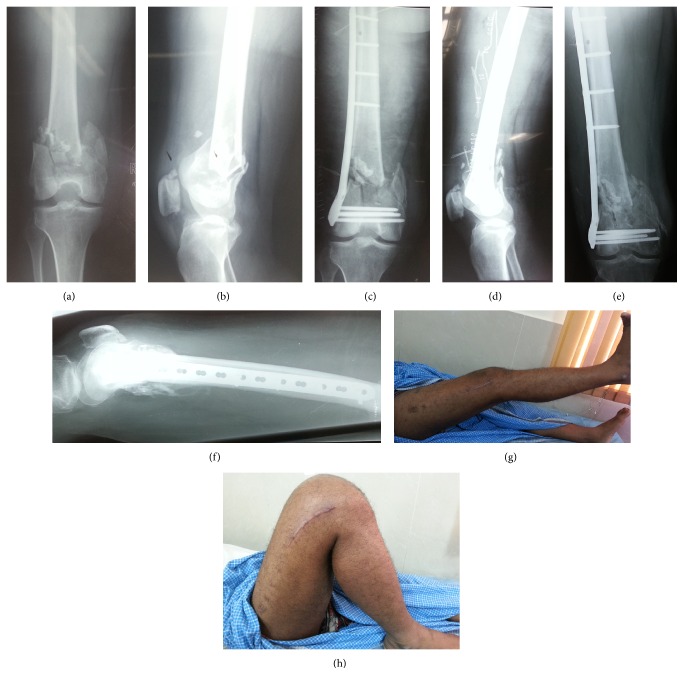
A 40-year-old male presented with an AO/OTA-type C2 supracondylar femoral fracture ((a) and (b)); the fracture was treated with AO distal femoral locking plate ((c) and (d)). The fracture healed with good alignment ((e) and (f)) with satisfactory knee movements ((g) and (h)).

**Figure 2:**
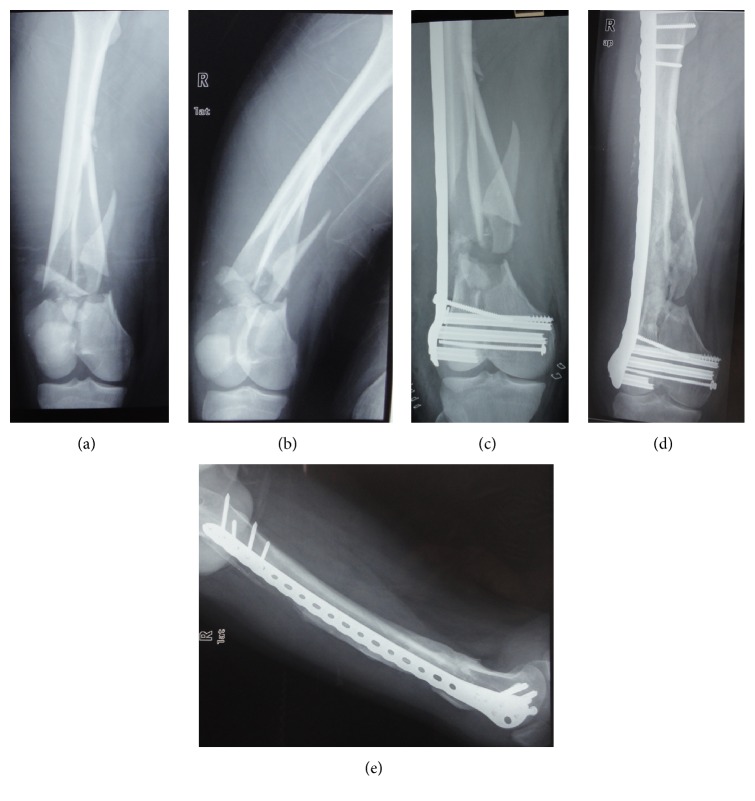
A-25-year old male patient with an AO/OTA-type C2 supracondylar femoral fracture ((a) and (b)); the fracture was treated with distal femoral locking plate (c) (Zimmer). The fracture healed with good alignment ((d) and (e)).

All surgeries were carried out at our tertiary care level 1 trauma centre with patient placed supine on the radiolucent table. A small towel bump was placed posterior to supracondylar region. A femoral distractor was used in 15 patients. Lateral approach was used for C1 fractures. Iliotibial band was incised along the line of skin incision. Vastus lateralis was retracted anteriorly to expose the distal femur. Anterolateral parapatellar approach was used in C2 and C3 fractures. Intra-articular fracture reduction was obtained and temporarily fixed with multiple K wires. Indirect reduction of articular surface with femoral diaphysis was done under fluoroscopic guidance. Appropriate-sized distal femoral locking plate was slid in distal to proximal direction submuscularly over lateral aspect of distal femur. Length of plate was determined intraoperatively after fracture reduction. Usually we prefer minimum length of the plate which is three times the fracture comminution segment. First a standard 4.5 mm cortical screw was placed in the femoral diaphysis and tightened lightly. For proximal fixation 3 or 4 bicortical screws were used percutaneously. Minimum of 5 locking screws were used for distal fixation (Figure 1). One or more partially threaded cancellous screws were used in intercondylar region whenever required to achieve compression (Figure 2). Position of the plate was confirmed under image in both AP and lateral views. No screws violated the intercondylar notch area. Small incision was given towards the end of the plate to confirm approximation of plate to the bone. Suction drain was used in 20 patients and was removed after 24 to 48 hrs. Primary bone grafting was not performed. Postoperatively limb elevation was given with knee in about 15 degrees of flexion. Static quadriceps exercises with active hip and knee mobilization were started from 1st postoperative day.

Postoperative radiographs were taken. Follow-up radiographs were taken after 6 wks, 12 wks, 6 months, 9 months, and 12 months after surgery. Initially nonweight bearing mobilization was started. Gradual weight bearing was started based on the evidence of bridging callous on follow-up radiographs. The average time until weight bearing was 3 months. Radiological union was defined as the presence of cross trabeculation on both AP and lateral radiographs. Nonunion was defined as failure of fracture union at 9-month follow up. Clinical and functional outcomes were assessed using the Knee Society score [8] (Table 2).

## 3. Results

**Figure 3:**
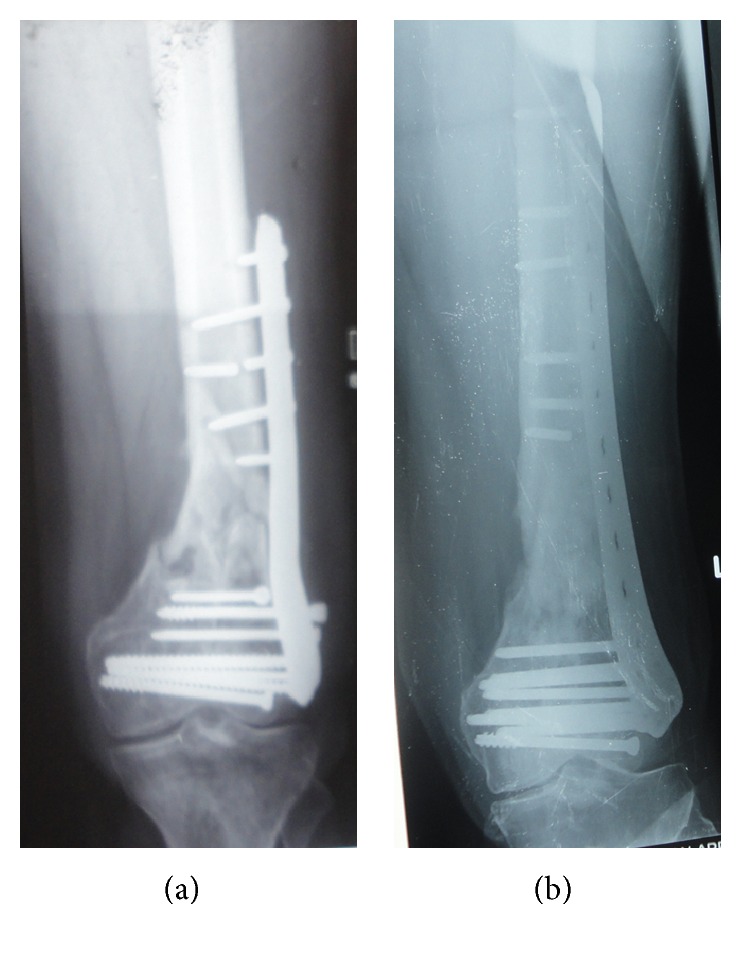
Nonunion of distal femoral fracture with fixation failure (a); union occurred after 3 months following refixation with longer plate and autogenous bone grafting (b).

The mean follow-up period was 12.3 months (mean 9–24 months). The mean time for radiological union was 14 weeks (range 8–18 weeks). There were 2 cases of nonunion. One case required autogenous iliac crest bone grafting and the other case required bone grafting along with refixation using longer plate due to breakage of proximal screws with broken proximal screws (Figure 3). Fracture went on to unite after 3 months of surgery. At latest follow up 38 patients had good/excellent outcome. 36 patients returned to their preinjury functional level.

One patient required arthroscopic arthrolysis with implant removal due to severe restriction of movement and hardware prominence. No cases of infection and rotational or angular deformity more than 5 degrees was noted (Table 1). 2 patients had limb length discrepancy of <2 cm and no treatment was needed.

At the latest follow up ROM > 120° is noted in 32 patients, 90–120 in 10 patients, and 70–90 in 2 patients.

## 4. Discussion

To maintain the fracture biology and to minimize the soft tissue trauma, minimally invasive plating techniques have been developed for the fixation of distal femoral fractures.

The main goals of the above-mentioned techniques are to maintain the important anatomy and to promote early fracture healing.

Locking plate systems such as the LISS [9] have been extensively used for distal femoral fractures [10–13]. LISS has a lower risk of early implant loosening than the dynamic condylar screw [14] and promotes early mobilization [15, 16] and rapid healing without bone grafting [15] with low risk of infection [15, 16] and less blood loss [16]. The LCP differs from the LISS in that the LCP has combination holes and does not have a jig [9]. Pain over lateral aspect of the distal femur following fixation with LISS has been attributed to the jig [13].

Previous studies have demonstrated successful early results and relatively low complication rates using minimally invasive plating techniques for the fractures of distal femur [12, 17, 18].

We have used minimally invasive plate fixation technique using standard lateral approach for the fixation of simple intra-articular fractures (C1). However, more extensive approaches are needed for fixation of complex intra-articular fractures (C2/C3). In these fractures we have employed lateral parapatellar arthrotomy for direct reduction of joint surface. This articular block was fixed to the femoral shaft using indirect plate fixation technique.

In our series of 44 patients, there were no cases of infection and varus/valgus alignment of more than 5 degrees. We noted 2 cases of nonunion, out of which one case required autogenous iliac crest bone grafting and the other case required bone grafting along with refixation using longer plate due to breakage of proximal screws. One case required arthrolysis with implant removal due to severe restriction of ROM and hardware prominence.

As reviewed by Miclau et al. [19] bone graft rates of supracondylar femur fractures ranged between 0% and 87%. Relatively low rate of bone grafting in our series is probably due to improved surgical technique with better soft tissue handling.

Early experience with the LISS for distal femoral fractures in multicentric study in Europe demonstrated a 20% incidence of varus/valgus deformity greater than 5 degrees [20]. Our relatively low incidence of deformity is probably because of improved surgical expertise along with better understanding of fracture anatomy.

Limitations of the study include (i) all the drawbacks pertaining to its retrospective nature and (ii) small sample size.

Strength of our study is that we included only intra-articular fractures; most of these were complex which require adequate surgical expertise.

The results of our study suggest that use of standard lateral approach for simple intra-articular distal femoral fractures (C1) and transarticular/minimally invasive techniques for complex intra-articular fractures (C2/C3) results in improved exposure of the knee joint and better union rates with low incidence of bone grafting. However randomized trials are needed to assess this technique.

## Figures and Tables

**Table 1:** Complications.

Complications	Number	Number requiring reoperation
(i) Infection	0	0
(ii) Nonunion	2	2
(iii) Varus/valgus deformity	0	0
(iv) Severe restriction of ROM	1	1

Total	3 (6.81%)	3 (6.81%)

**Table 2:** Type of fracture and outcome.

Type of fracture	Number	Knee society score (mean)	
		Knee score	Functional score
C1	10	96.5	92.9
C2	12	93.8	86.2
C3	22	86.1	79.3
